# Infant factors that impact the ecology of human milk secretion and composition—a report from “Breastmilk Ecology: Genesis of Infant Nutrition (BEGIN)” Working Group 3

**DOI:** 10.1016/j.ajcnut.2023.01.021

**Published:** 2023-05-10

**Authors:** Nancy F. Krebs, Mandy B. Belfort, Paula P. Meier, Julie A. Mennella, Deborah L. O’Connor, Sarah N. Taylor, Daniel J. Raiten

**Affiliations:** 1 Section of Nutrition, Department of Pediatrics, University of Colorado School of Medicine, Aurora, CO, USA; 2 Department of Pediatric Newborn Medicine, Brigham and Women’s Hospital and Harvard Medical School, Boston, MA, USA; 3 Department of Pediatrics, Rush University Medical Center, Chicago, IL, USA; 4 Monell Chemical Senses Center, Philadelphia, PA, USA; 5 Department of Nutritional Sciences, Temerty Faculty of Medicine, University of Toronto, Toronto, ON, Canada; 6 The Hospital for Sick Children, Toronto, ON, Canada; 7 Division of Neonatology, Department of Pediatrics, Yale School of Medicine, New Haven, CT, USA; 8 Pediatric Growth and Nutrition Branch, *Eunice Kennedy Shriver* National Institute of Child Health and Human Development, National Institutes of Health, Bethesda, MD, USA

**Keywords:** lactation, microbiome, milk removal, chemosensory, preterm milk, breastfeeding

## Abstract

Infants drive many lactation processes and contribute to the changing composition of human milk through multiple mechanisms. This review addresses the major topics of milk removal; chemosensory ecology for the parent–infant dyad; the infant’s inputs into the composition of the human milk microbiome; and the impact of disruptions in gestation on the ecology of fetal and infant phenotypes, milk composition, and lactation. Milk removal, which is essential for adequate infant intake and continued milk synthesis through multiple hormonal and autocrine/paracrine mechanisms, should be effective, efficient, and comfortable for both the lactating parent and the infant. All 3 components should be included in the evaluation of milk removal. Breastmilk “bridges” flavor experiences in utero with postweaning foods, and the flavors become familiar and preferred. Infants can detect flavor changes in human milk resulting from parental lifestyle choices, including recreational drug use, and early experiences with the sensory properties of these recreational drugs impact subsequent behavioral responses. Interactions between the infant’s own developing microbiome, that of the milk, and the multiple environmental factors that are drivers—both modifiable and nonmodifiable—in the microbial ecology of human milk are explored. Disruptions in gestation, especially preterm birth and fetal growth restriction or excess, impact the milk composition and lactation processes such as the timing of secretory activation, adequacy of milk volume and milk removal, and duration of lactation. Research gaps are identified in each of these areas. To assure a sustained and robust breastfeeding ecology, these myriad infant inputs must be systematically considered.

## Introduction

The “Breastmilk Ecology: Genesis of Infant Nutrition (BEGIN)” Project was designed to *1*) examine the ecology of human milk, based on the supposition that human milk represents a complex biological system that interacts with both the internal biology and health of the lactating person, the human milk matrix, and the impact on the breastfed infant and external (social, behavioral, cultural, and physical) environments (see Text Box 1 for Core Concepts and Terms); *2*) explore the functional implications of this ecology for both the biological parent and the infant; and *3*) explore the ways in which this emerging knowledge can be studied and expanded via a targeted research agenda and translated to support the community’s efforts to ensure safe, efficacious, equitable, and context-specific infant feeding practices in the United States and globally.

Text Box 1 **Core concepts and terms.**

• In the context of this paper, “ecology” is defined as a complex biological system and its interactions with its environment. In this case, the complex system is human milk composition and its inherent biology and the environment consists of parental and infant inputs and the influence of their respective internal and external environments.
• With due recognition of the need to be observant of issues of gender identity/neutrality and to improve precision, to the extent possible, for the purposes of the papers described herein, we will use gender-neutral terminology where appropriate (e.g., lactating parent/person, etc.) to reflect the reality that not all people who lactate identify as female. The term “lactating parent” respects and recognizes those who may have been born female but do not identify as such and other gender-relevant contingencies. In situations that report primary data (studies/analyses), we will refer to the population as specified (e.g., “the study evaluated 250 lactating mothers”). Moreover, rather than using terms such as “maternal” or “maternal milk,” we will use the terms such as “birthing parent” throughout the report as appropriate, as they accurately reflect the biological nature of the birthing parent-infant dyad.
• “Human milk” refers to milk produced by lactating parents and includes both (1) breastmilk produced by a parent for their infant that is fed directly to infants via the breast or expressed by the lactating parent and then fed to the infant and (2) donor/banked human milk produced by lactating persons that is either donated to human milk banks or fed to infants other than their own child.

Alt-text: Text Box 1

The overarching conceptual framework and description of the Project are presented in the BEGIN executive summary, the first of the 6 manuscripts of this supplement [1]. We hope that the reader will also review the subsequent manuscripts in this supplement, which present the findings of all of the individual thematic BEGIN Working Groups (WGs) as a continuum of thought that reflects a larger conceptual view of how we can move this important research and public health agenda forward.

Specifically, the BEGIN Project was accomplished by forming 5 thematic WGs charged with addressing the following themes: *1*) parental inputs to human milk production and composition; *2*) the components of human milk and the interactions of those components within this complex biological system; *3*) infant inputs to the matrix, emphasizing the bidirectional relationships associated with the breastfeeding dyad; *4*) the application of existing and new technologies and methodologies to study human milk as a complex biological system; and *5*) approaches to translation and implementation of new knowledge to support safe and efficacious infant feeding practices. As a subtopic of the BEGIN Project, this report (i.e., WG 3) broadly addresses biological processes driving the relationships between the infant and milk composition, secretion and release, and how these relationships are impacted by both parental and infant health and exposures. Knowledge gaps and areas warranting further study are identified.

**FIGURE 1:**
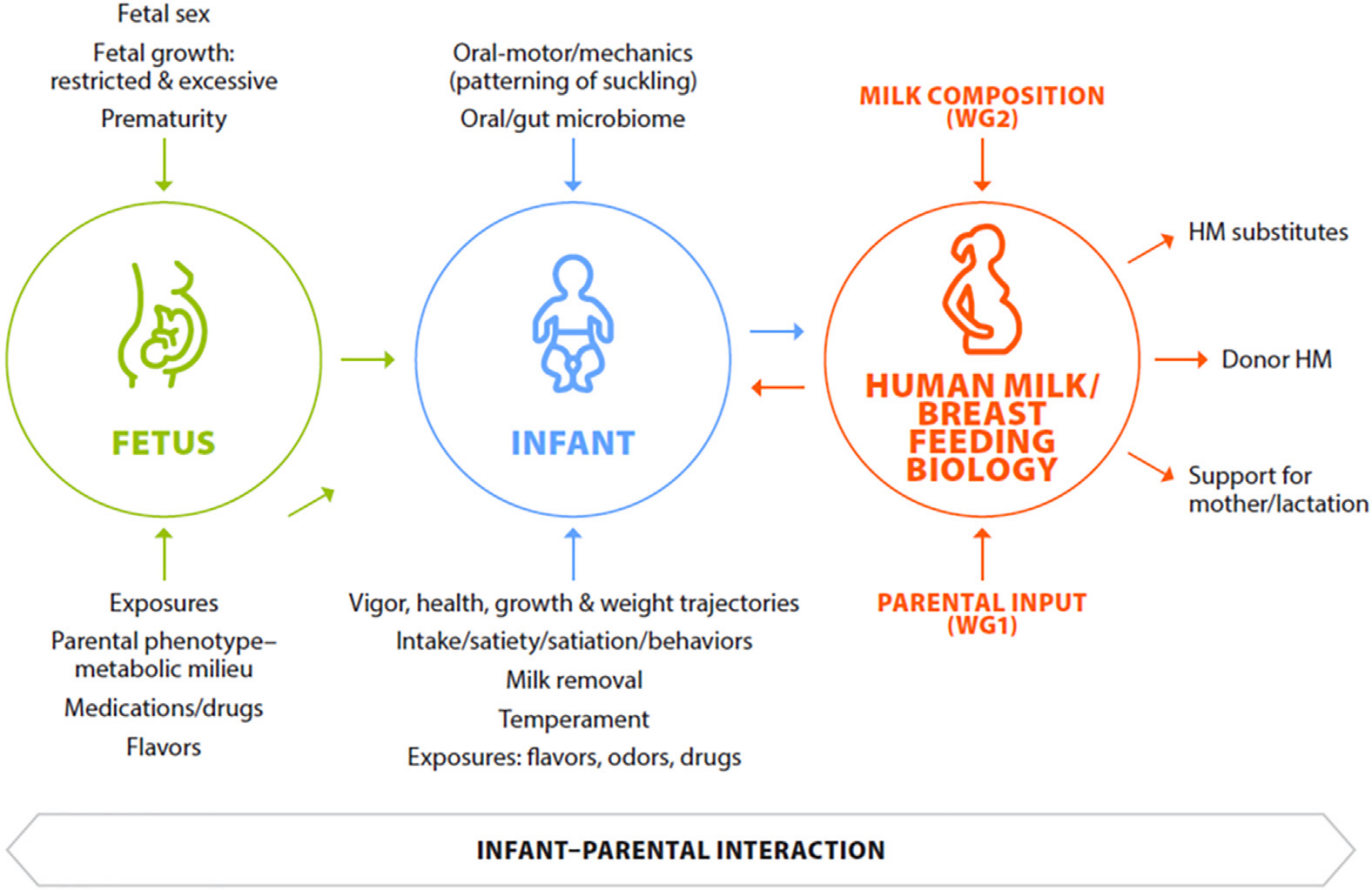
Conceptual framework for Working Group 3 discussion.

Far from being a passive recipient, infants drive many lactation processes and contribute to the changing composition of human milk through multiple mechanisms, including their innate genetic and phenotypic makeup (Figure 1). The infant contributes to human milk ecology primarily through milk removal and species-specific stimulation of the mammary gland, which are processes that assure adequate infant intake and regulation of the hormonal and autocrine/paracrine mechanisms that are critical to the initiation and maintenance of lactation [[2], [3], [4]]. In the healthy breastfeeding dyad, these processes are also affected by the shared sensory environment that begins in utero when chemical senses begin to functionally develop [2]. At birth, the newborn infant can detect, differentiate, behaviorally react, and learn via experiences with tastes, smells, and flavors in the context of the lactating parent and breastfeeding. Such postnatal sensory experiences, especially flavors, share continuity with amniotic fluid exposure; identify the lactating parent from others; and modify the infant’s milk removal, mammary gland stimulation, and satiation responses [5]. As such, breastmilk “bridges” flavor experiences in utero with postweaning foods, and the flavors become familiar and preferred.

The breastfeeding dyad also shares a highly individualized relationship via the human milk microbiome, which is an important contributor to the colonization of the infant’s gastrointestinal tract and oronasopharynx [6]. As also discussed in the report that addresses parental inputs [7], the diversity, richness, and composition of microbial communities in human milk reflect an ecology that consists of environmental exposures to both the infant and the lactating parent and reciprocal exchanges of microbes during suckling and milk removal. Alterations in the infant’s microbial colonization also occur during illness and environmental exposures such as medications, nutritional supplements, and food sources other than human milk.

Less is known about the impact on human milk ecology of disruptions and variations in gestation that affect the phenotype of the infant, including prematurity, fetal growth restriction, and excess fetal growth. Furthermore, these types of disruptions in gestation often reflect such underlying health complications in the birthing parent such as malnutrition, overweight and obesity, preterm labor, premature rupture of membranes, pre-eclampsia, placental disorders, and gestational or pre-existing diabetes. Such conditions in the birthing parent have been linked with delayed or impaired secretory activation, insufficient milk volume, and altered milk composition. In the context of these types of conditions, the breastfeeding dyad itself may be perturbed even when the infant is otherwise healthy.

At a foundational level, and with due regard to the myriad of influences on lactation biology and effectiveness that will be covered throughout this report, the infant’s primary role in the breastmilk ecology is milk removal. With that basic premise, the paper begins with current concepts regarding definitions, quantification, and mechanisms related to milk removal in the contemporary context. Subsequent sections of this paper highlight many of the infant inputs that contribute directly and indirectly to human milk ecology and identify relevant research gaps.

## Milk Removal: Infant Inputs as Links to Lactation Response

Milk removal during breastfeeding is accurately referred to as milk transfer or milk intake, and milk removal by a breast pump is accurately termed pumped milk volume. Milk removal is essential for adequate infant intake and continued milk synthesis through multiple mechanisms, including oxytocin release, suckling-induced prolactin secretion, and autocrine/paracrine regulation at the level of the individual breast [[2], [3], [4]]. Milk removal occurs through the combination of parental milk ejection (positive pressure) and infant or pump suction (negative pressure) [4]. Although milk removal is often used synonymously with milk production, milk volume, milk supply, and milk yield, these terms reflect only milk synthesis—not the transfer of milk to the infant or the breast pump collection kit, which is influenced by infant or pump inputs [8].

### Measuring milk removal

The key components of milk removal are effectiveness, efficiency, and comfort. However, central to using these components in research and practice is the understanding of the accurate measurement of milk removal during direct breastfeeding or breast pump use [9]. The clinical and research standard for measuring milk removal during breastfeeding is test-weighing, a procedure in which the infant is weighed in the same conditions prebreastfeeding and postbreastfeeding (clothing, pacifier, sensors, and tubing if in the neonatal intensive care unit [NICU]) on accurate digital scales [10, 11]. Each gram increase in postfeed weight is estimated to equal to 1 g of milk intake (1 g = 1 mL). Multiple studies have demonstrated the accuracy, feasibility, and acceptability of test-weights to measure milk intake in the home or in the NICU for a single feeding or over the course of days or weeks when performed by trained research staff, clinicians, and lactating parents themselves [[12], [13], [14], [15], [16], [17]]. While popularized as being less intrusive than test-weighing, observational tools such as the LATCH score (latching, audible swallowing, type of nipple, comfort level of the lactating parent, help in holding the infant to the breast) and clinical indicators such as audible swallows are not accurate measures of milk intake and should be avoided [13, 15, [17], [18], [19]].

Other accurate techniques to measure milk removal during breastfeeding have been reported, including deuterium dilution, wherein the administration of deuterium to either the lactating parent or the infant is measured in infant urine or saliva samples and thus serves as a marker of milk transfer over time [12]. The use of test-weights and deuterium dilution have recently been compared with respect to accuracy, feasibility, and acceptability by lactating parents [10, 13]. The pumped milk volume is measured volumetrically or by weighing prefilled and postfilled milk containers on an accurate scale [20].

### Standardizing “milk removal to available milk” in the breast

Studies that use milk removal as an outcome measure for research or quality improvement interventions often report only absolute volume differences regardless of the amount of milk that was available for removal at the time. Current research standards include measuring the percent of available milk removed (PAMR: 0%–100%), thereby standardizing the fact that 100 mL of milk intake may be 50% of available milk in one lactating parent and 90% of available milk in another [4, 13]. Although historical interest in the functional capacity of the breast dates back decades [21], research establishing an algorithm between computerized measurement of milk storage capacity and milk total fat provides an accurate estimate of PAMR, a technique that has been used in clinical studies with healthy and NICU populations [4, 12, 22, 23]. Clinically, the use of PAMR is promising for individualizing the daily frequency of milk removal that is needed to optimize milk synthesis while avoiding unnecessary pumping sessions for breast pump–dependent lactating parents.

**FIGURE 2:**
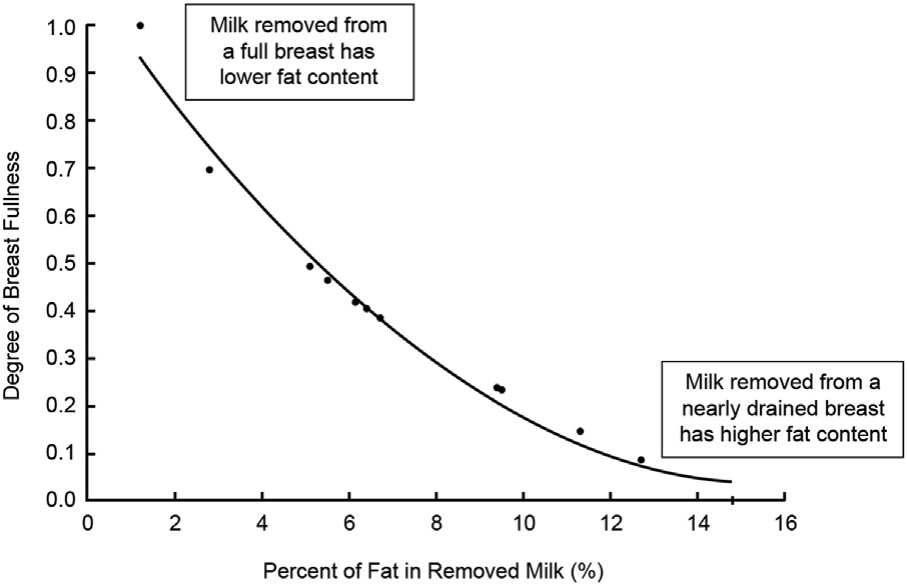
Relationship between the degree of breast fullness and the fat and caloric content (as % cream) of the removed milk, illustrating that caloric density is lowest when the breast is full and highest when the breast is nearly drained. Adapted with permission from Kent (2007) [4].

The importance of measuring PAMR is depicted in Figure 2, which reveals the within-parent variability in milk fat content as a function of breast fullness at the time of milk removal [4]. Milk fat, and thus caloric density, are lowest when the breast is fullest, and highest when the breast is nearly drained [4, 13]. Long intervals without milk removal result in high-volume, low-fat milk, whereas frequent milk removal results in smaller-volume, higher-fat milk [4, 13].

Although the healthy exclusively breastfeeding dyad balances the volume and fat content of milk intake over the day [24], breast pump–dependent parents of NICU infants provide individual milk collections that reflect this marked variability due to less regular milk removal [15, 25, 26]. Although individual collections have been reported to range from 18.9 to 34.3 kcal per ounce [25], most NICU default protocols assume that all parents’ own milk has 20 kilocalories per ounce and is fortified as such [27].

### Evaluating milk removal by the infant and breast pump

Milk removal should be effective, efficient, and comfortable for both the lactating parent and the breastfed baby, and all 3 components should be included in studies that evaluate milk removal during breastfeeding or pumping [9] (see Text Box 2).

Text Box 2 **Evaluation of milk removal—definitions.**

Effectiveness: As much of the available milk as possible is extracted. Influenced by both partners of the breastfeeding dyad (WG 1).

Efficiency: Available milk is extracted in a reasonable time frame (mL/min).

• Increases over time as lactation is established.
• Compositional change over the course of milk transfer.
• Fat content of the transferred milk increases by 3- to 12-fold with breast emptying (Figure 2) [4].
• Transfer of fat-soluble vitamins, cellular components, and odor-active volatile compounds concentrated toward the end of feed (WG 2)

Comfortable: Absence of breast or nipple pain in the lactating parent. Causes may be due to either partner of the breastfeeding dyad. For pump-dependent individuals, it may be due to poor quality pump, inappropriate suction pressures, poorly fitted breast shields, or lack of centering areola within the shield.

Alt-text: Text Box 2

Effective milk removal averages 67% for the breastfeeding infant and 62%–65% for a high-quality electric breast pump [13, 22]. As highlighted in the article describing parental inputs [7], effective milk removal reflects the regulation of prolactin and autocrine/paracrine mechanisms that are essential for continued milk synthesis [2, 3, 28].

Efficiency varies among breastfeeding dyads and stages of lactation [9, 13, 22]. During established lactation, infants transfer most of the milk during the initial 5 min of the feeds from the first (70%) and second (80%) breasts [29]. The fat content and lipid soluble compounds in removed milk increase as the breast empties [4, 21, 25, [30], [31], [32]] (Figure 2).

The early postpartum period is characterized by less-efficient milk removal by the breastfeeding infant or by pumping, especially during secretory activation when the available milk is limited [8]. As such, considerable time is required to remove even small amounts of milk during the early days postbirth. Intense stimulation of the breast during this critical early postnatal window may program the mammary gland’s structure and function such that lactation efficiency and capacity are optimized throughout lactation [2, 8, 9]. The concept of early intense mammary gland stimulation (as a function of limited milk availability) as necessary to optimize subsequent pumped milk volume was supported in a randomized, blinded study of breast pump–dependent parents of premature infants [33].

Comfortable milk removal has been evaluated in numerous studies of breastfeeding infants as well as in the context of breast pump use, and discomfort due to breast and nipple trauma can impact both effective and efficient milk removal and consequently may impact continued lactation. Examples of potentially problematic conditions include improper positioning during milk removal, mastitis, overweight or obesity in the birthing parent, flat or inverted nipples, “tongue-tie” (ankyloglossia) with poor attachment or nipple compression by the infant, or infant grasp of insufficient areolar area [34, 35].

### Infant sucking patterns during milk removal

The pattern of infant sucking consists of 2 components that alternate and are easily measured with noninvasive technologies: suction (negative pressure, mandibular opening) and expression (positive pressure, mandibular closure) [18, [35], [36], [37]]. Until the advent of ultrasound, the mechanism for milk removal during breastfeeding was thought to be expression, e.g., the infant’s compression of milk ducts to squeeze milk from the breast [37, 38]. More recently, ultrasound imaging revealed that milk transfer occurs during suction, whereas expression compresses milk ducts, stopping milk flow to enable safe swallowing [[39], [40], [41]]. These studies and others solidified the central role of infant suction in milk removal and underscored the findings of earlier researchers about the importance of suction in establishing and maintaining an effective milk removal position on the nipple [40, [42], [43], [44]]. Suction strength is developmentally dependent and is a primary reason for ineffective and inefficient milk transfer in premature, late preterm, and early term infants, as well as for infants with cardiorespiratory or neurodevelopmental disorders (see below) [45, 46].

### Infant sucking patterns during breastfeeding

Sucking is organized into bursts or runs of sucks (complete suction and expression phases) followed by pauses [36, 47]. Sucking rate refers to the frequency of sucks within a specific interval and sucking rhythm refers to the organization of bursts and pauses [36, 47]. Although sucking was initially characterized dichotomously as nutritive or nonnutritive, currently, a continuum of low-flow to high-flow milk is acknowledged [48, 49]. With high-flow rates, the infant must swallow frequently, which involves closing and reopening the airway, so sucking rates are slower and sucking pressures less intense. With low-flow rates, sucking rates increase and suction pressures are stronger [18, 39, 47, 50, 51]. Established breastfeeding is characterized by changing milk flow rates within a single feed, and the infant adjusts the sucking rate and rhythm to these changing rates, which, in turn, influences subsequent milk flow rates [13]. For example, rapid and strong sucks triggering the milk ejection reflex, which results in higher-flow milk, contributes to slower sucking due to swallowing. During secretory activation, when little milk is available, a low-flow pattern with rapid, strong sucks may be fundamental to programming milk synthesis [8, 9].

### Implications for design and use of breast pump technologies

Breast pump dependency has been defined as minimal, partial, or complete depending upon the extent to which the pump rather than the infant regulates the hormonal and autocrine/paracrine processes that are essential for the maintenance of lactation. This principle applies to all infants, regardless of whether they are preterm, late preterm, early term, or term [9]. Similarly, breast pump technologies vary with respect to effectiveness, efficiency, and comfort and can be matched to the needs of the breastfeeding dyad based on the extent to which the pump—rather than the infant—regulates lactation processes [9]. Examples of this classification scheme might include the following:

• Minimal: A parent who uses the pump only 2–3 times daily for convenience or separation is minimally breast pump–dependent and may tolerate a less-effective, less-efficient, or less-comfortable breast pump because their healthy infant compensates by regulating lactation processes during breastfeeding sessions for the remainder of the day.
• Partial: The infant, often born late preterm or early term, is capable of removing some of the milk from the breast but cannot feed with sufficient effectiveness or efficiency to regulate lactation processes. Continued breast pump use compensates for these weaknesses and is the primary regulator of lactation processes until the infant is able to do so.
• Complete: Parents who are unable to feed their infants directly or who elect to pump and feed their infants pumped milk by bottle are breast pump–dependent, meaning that the pump totally regulates lactation mechanisms. Most birthing parents of NICU infants are completely dependent on breast pumps for weeks or months until infants can remove milk effectively, efficiently, and safely, which often coincides with the infant’s expected birth date or even later.

Limited data suggest that breast pump–dependent parents become conditioned to the sounds and feelings of the breast pump similar to lactating parents becoming conditioned to the cries and sucking stimulus of the breastfeeding infant [22]. Thus, research focusing on the design of breast pumps and breast pump suction patterns that mimic the human infant during breastfeeding is a priority for both healthy and vulnerable populations [9]. The use of breast pumps may also be an important research tool to allow for the control of the effectiveness and efficiency of milk removal, which is not possible to regulate with a breastfeeding infant [52].

Selected research gaps related to milk removal are summarized in Text Box 3.

Text Box 3 **Effectiveness and efficiency of milk removal: selected research gaps.**

• What are the critical interactions of infant factors (e.g., prematurity, intrauterine growth restriction or excess, and infant suckling) with parental phenotype on milk removal and milk composition?
• What are innovations in accurate, minimally invasive measures of milk synthesis, removal, and standardization of measurements (e.g., milk biomarkers, imaging, and isotope dilution) that can be applied as point-of-care measurements in clinical and/or field conditions?
• How can breast pump technologies be optimized for effective, efficient, and comfortable milk removal for parents who have partial or complete dependence on mechanical milk removal? Do suction and flow patterns impact secretory activation and ongoing regulation of lactation?
• Do effectiveness and efficiency of milk removal impact the milk composition (e.g., nutritional, bioactive components, and xenobiotics)?
• What is the optimal pumping duration and frequency to support lactation and infant growth and development? Do these differ according to the stage of lactation and to the infant’s and/or parent’s clinical status? Can they be personalized to the individual parent’s milk storage capacity?

Alt-text: Text Box 3

## Chemosensory Ecology as a Birthing Parent–Infant Interface

Milk removal and species-specific mammary gland stimulation are exquisitely individualized within the breastfeeding dyad via bidirectional chemosensory signals that are exchanged between the lactating parent and the infant, and through the milk, as indicated in Figure 1. The biology of these relationships from the perspective of the lactating parent was discussed in the article describing parental factors [7]. The following sections focus on the implications of those relationships for the infant.

### Infant exposures, breastfeeding modulation, and chemosensory communication

At birth, the chemosensory ecology is composed of complex, 2-way interplays involving infants and lactating parents preferentially responding to the sensory features of the other [53] (see Text Box 4). Like amniotic fluid, human milk is a dynamic fluid with sensory properties that vary according to the health state and characteristics of the lactating parent [[54], [55], [56], [57], [58], [59], [60], [61], [62]].

Text Box 4 **Two-way interaction: summary of chemosensory exposures and effects**

1. Effects of sensory features of the birthing parent and their milk on the infant

• Recognition of and preference for the sensory features of the birthing parent
• Orientation and crawling toward the breast, nipple attachment, and initiation of feeding
• Pattern of suckling, infant nutrition, and growth
• Attachment and bonding
• Modulation of brain response to fearful stimuli
• Regulation of the behavioral state
• Providing information on dietary choices by the birthing parent. Diet-transmitted flavors in the amniotic fluid and human milk reflect the parent’s diet and provide flavor experiences that modulate later food acceptance
• Creation of flavor memories that modulate subsequent feeding

2. Effects of sensory features of the infant on the mother

• Recognition and preference for the sensory features of the infant
• Attachment, bonding, and coordination of care for the birthing parent
• Impacts on brain processing
• Modulation of hormonal responsivity and lactational process

Alt-text: Text Box 4

The functioning of the chemical senses of olfaction and taste, which are more mature at birth than other senses [63, 64], enables newborns to detect, differentiate, and behaviorally respond to a variety of chemical stimuli including the chemosensory features of the parent and the “taste” and flavor of human milk. These chemosensory stimuli provide entrainment with the chemosensory features of their intrauterine environment. Such continuity facilitates parental recognition, attachment, and bonding and affects the patterning of sucking, milk removal, mammary gland stimulation, and in turn infant nutrition [5, [48], [49], [50], [51], [52], 63, 64].

#### Body odors

Parental body odors, including human milk, have unique salience for their infants, and such chemosensory communication helps foster the transition from the fetal to postnatal environment [59, 63]. Shortly after birth and with little postnatal contact, the birthing parent can identify and prefer the olfactory signature of their newborns and vice versa [[60], [61], [62]]. When placed prone on their birthing parent’s abdomen, newborns will crawl toward the breast and prefer an unwashed to a washed breast [65, 66]—a source of lactating parental odors and one that shares similarity with the odor of amniotic fluid [67]. The body odors of the lactating parent, including those emanating from areolar glands [68, 69], and the taste and flavor of human milk [5] are identifying features that prime infants for breastfeeding and orient them to the breast, facilitate nipple attachment and suckling [63], and impact infant growth [70], and they can modulate responsivity to fearful stimuli [71] and overall behavioral state [69, 72, 73], even in the parent’s absence.

In addition to their impact on parental brain processing [74], many sensory features of the infant, including their body odors, modulate parental hormonal responsivity, which guides recognition and social interactions with the infant, including bonding [63, 75, 76].

#### Pain

Another fascinating aspect of the infant’s response to breastfeeding and human milk consumption is the impact that both of these have on an infant’s response to pain. An international body of research [[77], [78], [79], [80], [81], [82]] reports on the “analgesic” properties of sweet-tasting liquids for single events of pain following medical procedures (e.g., heel lance, venipuncture, and intramuscular injection) in neonates, infants, and children that appear to be, in part, mediated by endogenous opioid release [[82], [83], [84]]. In addition to its endogenous bioactive components, sweetness is a salient chemosensory property of human milk and is an important sensory component of breastfeeding. Breastfeeding during or shortly after a pain-producing procedure has been shown to reduce behavioral pain responses, and the modulating effect of breastfeeding on infant pain was more than that of each individual component (e.g., taste, smell, skin-to-skin contact, sucking, and taste or endogenous components of expressed breastmilk) [[85], [86], [87], [88], [89], [90], [91]]. Tasting expressed human milk was more effective than water [89] and was as effective as a sucrose-sweetened solution. Smelling their own parent’s milk was more effective than smelling the milk of an unfamiliar lactating parent, but both were more effective than the no odor condition [90] in attenuating pain responses. Although there is no research to date regarding the use of pasteurized donor human milk during painful procedures, expressed human milk and breastfeeding in general are safe and effective resources for the management of pain and can be used instead of oral sucrose [92].

### Lactating parent diet and other exposures

The flavor and composition of human milk change within and between breastfeeds and reflect what the lactating parent recently ate, drank, inhaled, and, to a lesser extent, applied to their skin [5, 93, 94]. In general, parental flavor ingestion and hence the flavoring of human milk are accepted by infants, as evidenced by increases in appetitive behaviors such as head orientation, mouthing, sucking, facial expressions of liking, and intake of human milk and similarly flavored foods and in arousal (e.g., increase in body movements) [5, 63, 64, [95], [96], [97]]. Interestingly, the recency of the flavor experience modifies the infants’ suckling response; suckling is more pronounced if infants had not experienced the flavor during the recent past [95, 96]. The flavor change may have aroused the infants; mammalian newborns will suck more [98] and exhibit a variety of other oral behaviors when aroused [97, 99]. Because both amniotic fluid and breastmilk reflect the dietary choices of the lactating parent, and breastmilk “bridges” experiences with flavors in utero and with foods the parent feeds after weaning. Infants are more accepting of foods containing flavors previously experienced in human milk [5, 64]. Thus, the flavor of the lactating parent’s diet becomes familiar and preferred by the infant over time.

### Lifestyle choices by the lactating parent: recreational drugs and infant exposures

The United States is in the middle of an epidemic of substance abuse, exacerbated by the recent COVID-19 pandemic [100, 101]. Lifestyle choices, including the most commonly used recreational drugs by pregnant and lactating parents—alcohol, nicotine, and cannabis (marijuana)—are on the rise, and there is an urgent need for evidence-based findings related to lactation and infant exposure, some of which are summarized herein. Additional perspectives on the impacts of these exposures are discussed by BEGIN WG 1 [7] and WG 5 [102].

### Transmission of xenobiotics to human milk and consequences of infant exposure

As addressed in the WG 1 report [7], important determinants of the concentration of a substance in human milk are its concentration in the lactating parent’s plasma (nonprotein-bound) and the interfeed and intrafeed blood flow to the breast [103]. This applies to xenobiotics, which include environmental agents such as medications, recreational and illicit drugs, pesticides, and endocrine-disrupting chemicals. For alcohol and tobacco, infants can detect flavor changes in human milk [104, 105], and early experiences with the sensory properties of these recreational drugs impact subsequent behavioral responses during childhood [[106], [107], [108]]. Whether similar flavor changes occur following cannabis or other drugs is currently unknown.

Extensive evidence indicates that use of alcohol, tobacco, and cannabis by a lactating parent, alone or in combination, impacts the developing brain and is linked to adverse pregnancy outcomes, adverse outcomes in infants and children, and adverse academic performance and drug misuse in adolescents [[109], [110], [111], [112], [113], [114], [115], [116], [117], [118]]. The contributions of drug exposure via human milk alone and how breastfeeding may protect against such exposures [119] are often confounded by the reality of prenatal exposure and that polydrug use is common [120, 121]. Many parents who use these drugs have partners or other household members who do use drugs as well [122], thereby increasing the likelihood of infants’ secondhand exposure to tobacco and cannabis [123]. The following sections highlight the challenge of distinguishing the direct effects of the drug on the parental lactational performance from the effect on the recipient infants.

#### Alcohol

Despite considerable research on the effects of prenatal alcohol exposure, data on the effects of postnatal alcohol exposure are limited. A rich folklore passed down the generations (including by health professionals) contended that drinking alcohol facilitates milk ejection, rectifies milk insufficiency, and calms “fussy” breastfed babies [124]. Experimental research called the lore that drinking alcohol benefits lactation into serious question [104, 114, [124], [125], [126], [127], [128], [129], [130], [131]]. When lactating parents who had abstained from drinking alcohol for at least 3 d drank 1–2 standard drink equivalents, alcohol was transferred to their milk in amounts almost identical to plasma, peaking within the first hour after ingestion and decreasing thereafter [104]. On average, the dose delivered to the infant ranged from 2 to 10 mg/kg of body weight [104]. The altered flavor of human milk paralleled the changes in human milk ethanol concentrations [104, 132]. During the breastfeeds that occurred within hours of alcohol ingestion by the lactating parent, infants’ behaviors were altered. They sucked more frequently from the breast during the first few minutes of the feedings, ingested less human milk [104, 132], and had less active sleep [128]. During the next 24 h, infants compensated for the changes in intake and sleep [128, 133].

Alcohol ingestion by the lactating parent disrupts the hormonal milieu (prolactin increases, oxytocin decreases), delays milk ejection, and reduces milk availability in the short term [130, 134]. While infants could detect the alcohol flavor in human milk, they did not reject it [126]. The quantity of alcohol ingestion by the lactating parent during lactation modified the infant’s patterning of suckling to its flavor in human milk by increasing sucks per burst and pauses [104, 126]. The effects apparently persisted, as children learned and retained sensory information about alcohol and formed emotional associations with how often and why their parent drank [106, 107, 135]. This body of research highlights the complexity of assessing whether changes in infant behaviors are due to the alcohol’s effect on the hormonal milieu of the parent, due to a direct infant response to alcohol-induced changes in human milk (e.g., flavor) [104], or due to the changes in the parent’s interaction with the infant [128, 136].

#### Tobacco

Nicotine is transmitted in a time-dependent manner to human milk, and exposure modifies the recipient infant’s behavior in the short term [105, 137]. When lactating parents who abstained from smoking for at least 12 h smoked 1–2 cigarettes, nicotine levels peaked within an hour of smoking and decreased thereafter [101]. The dose of nicotine delivered to infants was ∼550 ng/kg. Cotinine, the major metabolite of nicotine, was 10-fold higher in the urine of human milk–fed infants of smoking lactating parents when compared to bottle-fed infants of smoking lactating parents, suggesting that the primary mode of exposure is through human milk [123]; nicotine concentrations in milk were associated with pronounced changes in its flavor [105]. While no differences in human milk intake occurred during feeds within 3–4 h following parental smoking, differences did occur in infants’ sleep–wake patterning [128, 138]. Breastfeeding from parents within hours of smoking resulted in infants spending less time in active sleep and waking from their naps sooner; greater doses of nicotine delivered to the infant were associated with less time spent in active sleep [139].

#### Cannabis

To mitigate unintended consequences of marijuana legalization on infant development, medical authorities have issued calls for more research on the safety of cannabis use by lactating parents, particularly how exposure to cannabinoid-containing amniotic fluid or human milk affects infant development [109, 110, 140, 141]. Indeed, lactating parents frequently ask their healthcare providers whether cannabis use has an impact on fertility and/or poses risks of harm to the fetus or to the human milk–fed infant [142].

Limited scientific evidence is available regarding the transfer of cannabis to amniotic fluid and human milk following ingestion, inhalation, and vaping, especially with currently used products [109, [143], [144], [145], [146]]. While cannabis contains hundreds of chemicals, the fat-soluble tetrahydrocannabinol (THC)—a psychoactive component of cannabis—is the chemical most often measured. After a 24-h abstention, parental inhalation of 0.1 g of cannabis containing ∼23% THC resulted in its transfer to human milk [147]. The concentration of THC peaked 1 h postinhalation and decreased steadily in the next 4 h. The calculated dosage that would be delivered to an exclusively breastfed infant would be ∼8 μg/kg/d [148]. In a prospective observational study of 7 females who reported using marijuana during the prenatal period and who agreed to abstain for a period of 6 wk, blood and milk samples were collected 2–5 times per week. The half-life of THC in human milk was 17 d, with a projected time to elimination of 16 wk [148]. Smoking cannabis resulted in the transfer of THC to human milk that was 7.5 times higher than transfer to plasma [149, 150].

Pharmacokinetics data on the transfer of THC and other active cannabinoids to human milk following inhalation, ingestion, or topical application are scant. Among human milk–fed infants of chronic and heavy marijuana users, THC was found in both feces and urine [149].

Data on the effects of marijuana use and infant input are limited, conflicting, and outdated [109, 110, 151]. A study in 1985 [152] reported no effects of marijuana use by lactating parents on infant growth or development at 1 y, whereas in 1990, others [116] reported slight deficits in motor development but not growth or intellectual development if cannabis was used for more than 15 d during the first month after birth. The early experimental findings on transmission to human milk and consequences in the infants should be interpreted cautiously because cannabis legalization has resulted in a wider variety of products for ingestion, topical application, or inhalation/vape, and the potency is at least 300% greater than that of products used decades ago [153]. The prolonged time to elimination challenges the advice given to lactating parents regarding cannabis use during breastfeeding [154], and research is needed to determine the impact of such prolonged exposure on sleep, feeding, and cognitive development of the recipient infants.

Selected research gaps related to milk removal are summarized in Text Box 5.

Text Box 5 **Chemosensory ecology as a parental-infant interface: selected research gaps.**

• How do the chemosensory properties (e.g., volatile constituents) of human milk affect infant feeding behaviors, growth, sleep, and neurodevelopment, both short-term and long-term?
• How can new analytical tools detect chemosensory properties of milk?
• What are the pharmacokinetics of xenobiotic and bioactive components in milk? Are there effects on quantity and quality of milk production, and what are the concurrent effects on the infant?
• What are the pain-mitigating mechanisms and constituents experienced by the infant? Are there dose responses of candidate compounds on the quantitative and qualitative infant reaction to adverse stimuli?
• Does the chemosensory ecology of human milk and lactation differ between well-nourished and malnourished parents and their infants?

Alt-text: Text Box 5

## The Human Milk Microbiome as a Reflection of Infant Inputs

As addressed in the WG reports focused on milk composition and parental inputs [7, 155], an important component of the human milk ecology is its microbiome. The human milk microbiome, by definition, consists of both microbes (e.g., bacteria, viruses, and fungi) and their genes in human milk. In addition to parental inputs, the breastfeeding infant also contributes to breastmilk composition by its influence on the human milk microbiome, although the exact mechanisms have not been fully elucidated. Historically, it was believed that human milk was sterile [156] and that bacteria, fungi, or viruses detected in milk were attributed to localized or systemic infection in the lactating parent (e.g., mastitis and HIV) or contamination from a caregiver’s skin (e.g., breast, and hands), nasal secretions, or milk collection and storage devices (e.g., pumps). Contemporary evidence suggests that human milk, like other different sites in and on the human body (e.g., vagina, oral cavity, and gut), contains a distinctive innate microbial community and their genetic material. The following sections highlight the interactions between the infant’s own developing microbiome, that of the milk, and the multiple environmental factors that are drivers, both modifiable and nonmodifiable, in the microbial ecology of human milk.

### Infant factors affecting the microbiome of human milk

**FIGURE 3:**
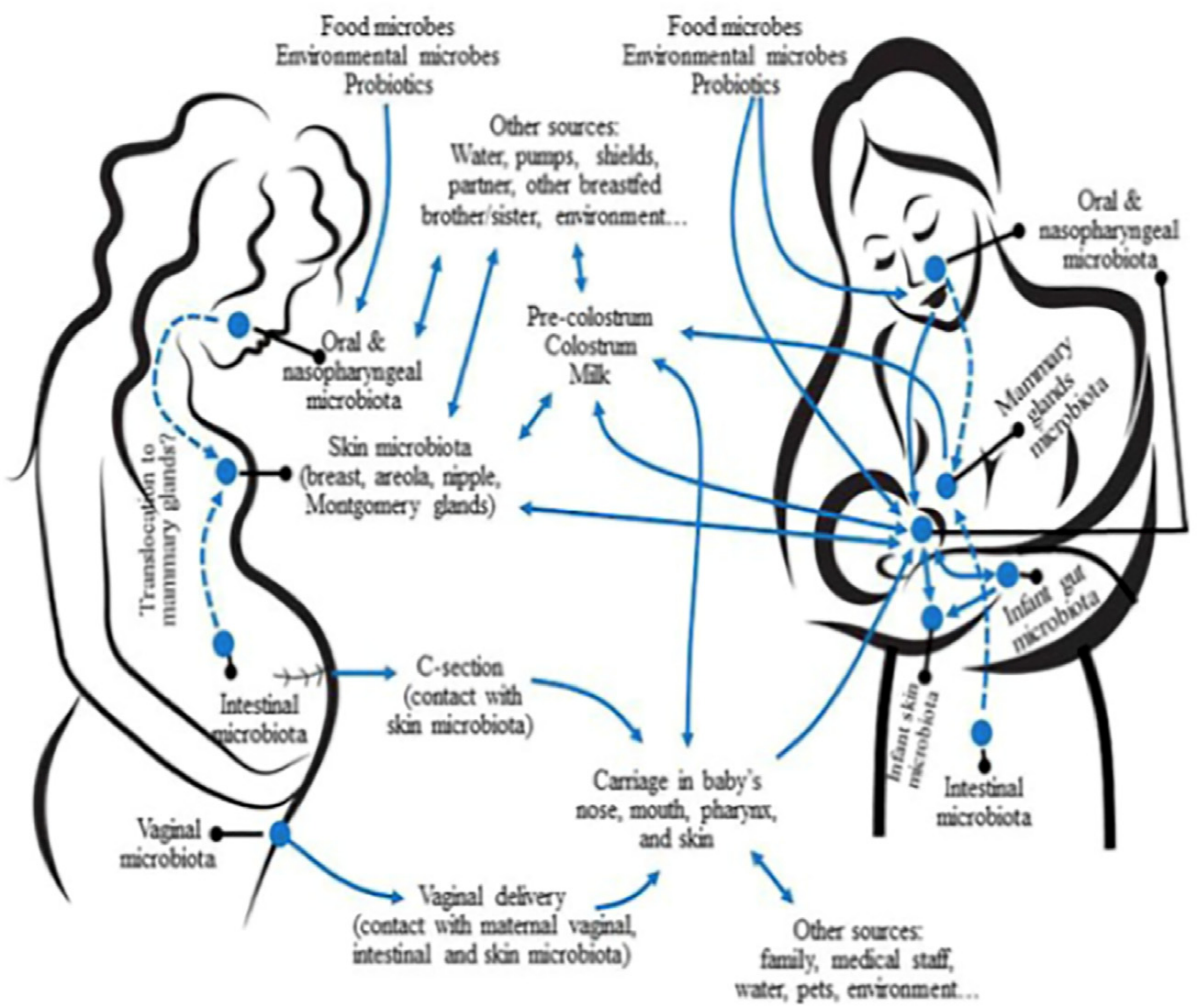
Potential sources of microbes present in human milk and interactions with other lactating parent-infant microbiota. Dashed arrows represent potential translocation through an endogenous pathway. Reproduced from Fernandez et al. [217] under the terms of the Creative Commons Attribution License (CC BY).

Most studies to date have focused on lactating parents’ factors influencing the human milk microbiome; however, the role of the infant in shaping the milk microbiome has recently generated significant interest. One mechanism by which microbes make their way into human milk is via an infant’s oral cavity, from which the microbes migrate to mammary gland ducts through retrograde transfer during direct breastfeeding (Figure 3). This evolving understanding of the contributions to the human milk microbiome is supported by observations of the differences in milk microbiota diversity and/or abundances of bacteria and predicted microbial functions in human milk collected from parents of term-born infants fed directly at the breast vs. those providing pumped human milk [[156], [157], [158]]. Also consistent with this understanding is the lower overall relative abundance of *Streptococcus*, a genus of bacteria common in the oral cavity, in human milk from lactating parents of tube-fed preterm infants than the literature values of healthy infants who were directly breastfed [157, [159], [160], [161], [162], [163]]. Introduction of complementary or solid foods to the diet of the normally developing healthy term infant impacts the microbial profile of their gut [164, 165]; for example, adult-associated microbes become more prominent. Whether the timing of introduction of complementary feeds could influence the microbial composition of human milk by direct breastfeeding and retrograde transfer is unclear.

Whether, and the degree to which, changes in the human milk microbiome are mediated by the infant’s own microbiome, by the dynamic interplay between the lactating parent’s microbiome and that of the infant, or by some other mechanism is not entirely clear.

Other evidence linking infant characteristics and the human milk microbiome include infant sex-dependent associations between breastfeeding mode, BMI of the birthing parent, and delivery mode (e.g., vaginal birth and cesarean section), among others [157]. While being an exciting area of research due to its potential clinical implications, the early stage and observational nature of this work leaves uncertainty regarding whether the observed associations are mediated by other factors such as milk synthesis or factors related to the parent, such as illness (e.g., antibiotic use and medical condition) or caregiving practices, which could differ by the sex of the infant.

### Implications of infant conditions for the human milk microbiome

#### Infections

Active infection in infants from a variety of ailments, including influenza, measles, and respiratory and gastrointestinal infection, has been associated with an increase in human milk leukocyte concentrations, which quickly return to baseline after recovery [166, 167]. While the mechanisms for this response are unclear, it is plausible that active infection in the infant either co-occurs with or results in an infection of the parent and/or that the presence of infant infection signals the need for an inflammatory lactating parent response. Both mechanisms result in increased secretion of white blood cells and cytokines into milk, a cascade of events that could potentially affect the microbiota in human milk.

While the impact of antibiotic use by the lactating parent on the human milk microbiome has been recently reported by a number of research groups [160, 168, 169], the impact of infant antibiotic use on the human milk microbiome has largely been unexplored. It is well known that antibiotic use during infancy alters the gut microbiome of the infant [164, 170, 171]. Whether an altered infant gut microbiome can have a clinically relevant impact on the human milk microbiome remains to be determined.

#### Gastric acid suppression and gastroesophageal reflux

Some evidence suggests that the use of proton pump inhibitors as therapy for gastroesophageal reflux disease may also be associated with changes in an infant’s microbiome, including microbial diversity and relative abundances of some microorganisms [161, 162, 172, 173]. The impact of proton pump inhibitor use in an infant on the human milk microbiome composition is unknown. Hypothetically, an altered infant microbiome could be transferred to the mammary gland, but this remains to be confirmed.

#### Preterm infant and human milk microbiome

Prematurity and the preterm infant’s unique environment have significant implications for the development of the infant’s microbiome. Microbial colonization of the gastrointestinal tract is either associated with or perturbed in the very low birth weight (VLBW; <1500 g) infant by several factors, including cesarean delivery, widespread antibiotics use, parent–infant separation, prolonged rupture of the membranes, delayed enteral feeding, tube feeding, micronutrient supplementation, and living in a NICU populated with an enriched pathogen load [[174], [175], [176]]. Perturbations of the microbiome may include blooms of potential pathogens, loss of protective commensals, or loss of diversity; such effects may be generically termed “dysbiosis.” Strategies adopted to mitigate clinically significant imbalances in the gut microbiome include antibiotic stewardship, feeding fresh vs. previously frozen human milk, use of donor milk vs. formula as a supplement to parent milk, routine use of probiotics, oropharyngeal colostrum administration, and “skin-to-skin” care, a practice where a parent or caregiver holds the diaper-clad but otherwise naked preterm infant to their own bare chest routinely [177, 178].

As described earlier, the microbial composition of milk collected from parents of term vs. preterm infants is known to differ [160, 161, 179]. Whether and to what extent “dysbiosis” in the preterm infant contributes to differences in milk composition requires systematic investigation, as do associations between strategies to mitigate dysbiosis and milk composition. Disentangling the directionality of associations between parent and infant microbiomes and the potential cumulative impact of possible contributors to the human milk microbiome is challenging because it is likely that these relationships are complex, interrelated, and bidirectional. Selected research gaps related to infant inputs to the human milk microbiome are summarized in Text Box 6.

Text Box 6 **Infant inputs to the human milk microbiome: selected research gaps.**

• Can favorable microbial patterns (diversity and composition) in human milk that promote infant health and development be identified? Longitudinal characterization of infant responses according to the microbiologic profile of human milk consumed is required.
• Evaluation of the relationship of different microbial profiles associated with human milk oligosaccharide composition and infant functional outcomes (e.g., growth, development, and morbidity) is required.
• Are the differences in the microbiome of human milk associated with functional differences in infant development? Longitudinal assessment of infant neurodevelopmental responses to different milk microbiome profiles is required.
• Does the infant’s sex promote differences in milk microbiome, independent of the birthing parent’s diet and nursing or feeding patterns? Twin studies to compare the effects of infant’s sex on milk composition, including microbiome, independent of birthing parent factors (e.g., diet and habits) may be promising.
• What are the impacts of infant medically directed exposures in hospital vs. in home on the infant’s enteric microbiome and that of milk? Prospective investigation of the relationship of infant’s enteric, skin, and oral microbiome on the microbial composition of milk is warranted.
• Test effects of infant micronutrient supplementation on enteric and milk microbiomes, ideally employing a randomized controlled trial (RCT) design. Test effects of infant prebiotic or probiotic administration on enteric and milk microbiome, ideally employing a randomized, controlled trial (RCT) design.
• What is the impact of preterm birth vs. term birth on the infant microbiome. Investigations suggested above conducted in the context of differences in infant gestational age.

Alt-text: Text Box 6

## Disruptions in Gestation: Ecology of Fetal and Infant Phenotypes, Milk Composition, and Lactation

Disruptions in gestation affect the phenotype of the fetus and newborn and impact the ecology of human milk, including both milk composition and lactation processes such as the timing of secretory activation, adequacy of human milk volume, milk removal (by infant or breast pump), and duration of lactation (Text Box 7). The effects of infant conditions may add to, or interact with, the effects of health problems in the birthing parent and/or complications of pregnancy and delivery, which also contribute to the differences in human milk composition and lactation processes. Furthermore, parents of these infants are more likely to rely on lactation technologies when infants are unable to feed directly from the breast. The implications for the ecology of human milk by these interacting factors are referred to in Figure 1 and explored in the following sections.

Text Box 7 **Factors influencing the adequacy of human milk for the preterm infant.**

1. Interruption of fetal (parenteral) nutrient supply.
2. Ongoing milk production dependent on regular and sustained pumping.
3. Infant only tolerates feeding volume less than that produced.
4. Concentrations of some nutrients in preterm milk are higher than those of term milk; elevations may not be sustained.
5. Dynamic changes in nutrient concentrations over weeks postpartum may be out of sync with infant’s requirements.
6. Nutrient absorption, utilization, and retention are impacted by immature organs (gut, kidneys), contributing to increased requirements.

Alt-text: Text Box 7

### Preterm delivery

Compositional differences in the human milk of parents delivering preterm infants have been addressed separately [155]. To date, the mechanisms for these differences have not been definitively delineated. Preterm delivery is associated with a delay in the onset of secretory activation, low milk volume [20], and altered milk composition including that of both nutrients and nonnutrient bioactive factors. Several factors contribute to these differences. A shortened gestation means less time for prenatal preparation by the mammary gland, although how preterm delivery interrupts mammary gland development and how this affects milk composition are not well studied. After delivery, the preterm infant exhibits immature oral feeding skills, meaning that milk removal is impaired or absent. This prompts the need for dependence on alternative methods of milk removal such as manual and/or mechanical breast pump use, which may independently contribute to both delayed secretory activation and insufficient milk volume.

Preterm infants are often unable to achieve effective and efficient milk removal to meet their nutritional needs and to maintain lactation processes. This is due to not only immaturity in oral motor skills but also an inability to communicate hunger (e.g., waking to feed, rooting, and sucking on hand) or satiety (e.g., pulling off nipple and turning head). The ability to feed orally develops gradually and is typically in place by 36–42 wk of postmenstrual age, with the timing inversely related to the gestational age at birth and with possible further delay due to other clinical conditions. For preterm infants with cardiorespiratory disease, compromised endurance complicates the development of oral feeding abilities. Neurological sequelae of preterm birth can further delay the maturation of oral feeding.

Factors shown to relate positively to the infant’s development of the ability to remove milk effectively and efficiently from the breast include an abundance of available milk in the breast; skin-to-skin or “kangaroo” care; feeding directly at the breast before bottle feeding; and shorter duration of neonatal hospital stay [180, 181]. The clearest evidence regarding methods to optimize preterm infant feeding directly from the breast are studies of kangaroo care and the use of test-weighing [14, 16]. In a randomized, controlled trial in a population of infants born at 32–36 wk of gestation, unlimited kangaroo care was associated with a longer duration of breastfeeding (5 vs. 2 mo) and longer exclusive breastfeeding [182]. In a case-control study, mean skin-to-skin time (in min) per day was higher in lactating parent–infant dyads who sustained breastfeeding through 6 mo compared with that of those who did not [183].

#### Alternative modes of human milk feeding of preterm infants

With impaired or absent breastfeeding, the parent or care team is dependent on alternative methods of milk removal; milk is then fed to the infant via gavage tube or other oral means. Optimal timing from the delivery to the initiation of breast pump use among parents delivering VLBW infants has been the focus of recent observational and interventional studies. In contrast to the early studies demonstrating benefit with the initiation of breast pump use as soon as possible after very preterm delivery, newer evidence suggests that initiation of milk removal within up to 6–8 h postdelivery may be equivalent to earlier removal [181, 184, 185]. Newer evidence also points to breast pump use 5 times per day as adequate once the milk volume has been established, and to the maximum interval between pumping sessions as not greater than 7 h [186].

Even though very preterm infants have limited oral skills, during kangaroo care, suckling on an empty breast appears to be safe relative to aspiration risk [187], exposes the infant to the tastes and smells of the parent’s milk [32], and may contribute to improved milk synthesis and provide infant input to the milk microbiome. After 32–34 wk of gestation, actual oral feeding from the breast or bottle may begin, but the infant is typically not able to remove sufficient amounts of human milk until the infant is closer to term, and perhaps longer, especially if the infant’s development and/or stamina is limited by cardiorespiratory and/or neurologic diseases.

#### Milk composition

As highlighted in other reports from the BEGIN WGs 1 and 2 [7, 155], numerous human milk components differ between preterm and term newborns, yet the underlying drivers of these differences remain largely unknown. Despite higher levels of protein and some other nutrients, human milk alone is inadequate to meet the infant’s relatively high nutrient requirements, particularly for infants born very preterm (<32 wk of gestation) and extremely preterm (<28 wk of gestation). When comparing human milk expressed for preterm infants with the milk expressed for term-born infants, preterm milk has been shown to differ in macronutrient, micronutrient, and bioactive composition. In a meta-analysis of the composition of 24-h milk collection, preterm milk was higher in fat and true protein in the first week, but then levels decreased to concentrations similar to term milk at 4 wk and 10–12 wk, respectively [188]. Calcium was lower through the first 4 wk and then was higher beyond 4 wk [188]. Although the total fat content varied between preterm and term human milk, fatty acid patterns were similar [189]. Bioactives shown to differ between preterm and term milk include human milk oligosaccharides, antioxidants, hormones/growth factors, adipokines, inflammatory and anti-inflammatory cytokines, cells, and microbes [[190], [191], [192], [193], [194], [195], [196], [197], [198]].

Milk glucocorticoid concentrations appear to relate to the degree of prematurity, which raises the question of whether this source of increased cortisol is related to preterm birth itself or to the stress related to preterm birth [199]. To further magnify the complexity in the investigation of how milk bioactive concentrations differ with preterm birth, human milk protein concentrations vary by the length of antenatal steroids given during pregnancy to improve preterm infant outcomes [200]. Therefore, milk composition may not only differ with preterm birth but also by medical intervention, parent wellbeing, and gestational disease.

#### Implications of lactation challenges consequent to preterm birth

By necessity, there is a mismatch between the optimal daily pumped milk volume for the parent to maintain lactation processes (≥500 mL) and the infant’s daily feeding/intake, which may be only 20%–75% of the daily pumped milk volume. This mismatch likely perpetuates nutritional deficits for preterm infants receiving parent’s milk, since high concentrations for some nutrients do not compensate for the very small volumes ingested, particularly in relation to the high requirements. Additionally, with preterm birth, a portion of fetal (parenteral) nutrient delivery is inevitably missed, and parent’s milk nutritional composition does not necessarily surmount these deficits. Immature organ systems contribute to higher nutrient requirements via impacts on absorption, utilization, and retention. Parent’s milk may not meet phosphorus, calcium, protein, energy, and zinc needs of preterm infants. The capacity for desaturation and elongation of long-chain polyunsaturated fatty acids is limited due to immature enzyme systems. Meta-analysis of multicomponent human milk fortifier supplements to human milk demonstrates improved anthropometrics, but evidence is lacking regarding long-term outcomes such as improved neurodevelopment or bone mineralization when compared to infants receiving human milk without fortification [201].

### Fetal growth restriction

Compared to the impact of prematurity, even less is known about how gestational disorders leading to fetal growth restriction, such as pre-eclampsia and other forms of placental insufficiency, affect the milk composition and infant milk removal behaviors. Small-for-gestational age (SGA) infants have greater nutritional needs at birth and often exhibit catch-up growth after birth. In some existing studies, human milk macronutrient and fatty acid profile composition do not differ from human milk for appropriate-for-gestational age (AGA)–weight infants [[202], [203], [204]]. Therefore, the postnatal catch-up growth exhibited by intrauterine growth restricted (IUGR) infants may be related to the volume of human milk intake rather than compositional differences. Yet, other studies have described lower human milk intake and lower fat and protein density in SGA infants demonstrating poor infant growth [205]. In an animal model of placental insufficiency—often an underlying cause of fetal growth restriction—mammary development appeared similar, but milk nutrient delivery, as measured by calcium transport, appeared reduced [206]. Postnatal growth in term-born infants results from increased food intake, which is promoted through resistance to leptin effects and increased sensitivity to appetite-stimulating hormones such as ghrelin. These hormones are present in the human cord blood and milk and may play a role in dyadic signaling during breastfeeding [207, 208]. The impact of these hormonal factors in preterm or term milk on postnatal growth in infants who have experienced intrauterine growth restriction is unclear.

Metabolomic evaluation has shown differences in milk metabolites between birthing parents with pre-eclampsia and birthing parents serving as controls at day 3 and continuing through 6 mo [209]. Some metabolomic differences occur in the phosphocholine and glycerophosphocholine pathways, which influence protection against oxidative stress and brain development [210].

#### Hunger-satiety signaling

The dyadic communication between a parent and the full-term, “on-demand”–fed infant is central to the synchronization of individualized milk synthesis and milk removal via autocrine or paracrine mechanisms based on the infant’s nutritional requirements for growth and development [2, 211, 212]. Infant hunger-satiety signaling is critical to maintain the appropriate balance of infant intake and available milk. Animal models demonstrate the mechanisms by which fetal growth restriction may program postnatal appetite control, which occurs mainly through the effects on 2 neuronal cell types within the hypothalamic arcuate nucleus [213].

### Fetal growth excess

Fetal growth excess leading to a large-for-gestational age (LGA) newborn phenotype is most often related to parent gestational or pre-existing diabetes, insulin resistance, and/or obesity. These gestational complications are associated with delayed initiation of secretory activation, which may be related to immature feeding patterns, reliance on breast pump use, or also potential mammary gland abnormalities. Interestingly, LGA infants and infants exposed in utero to diabetes in the birthing parent may exhibit immature sucking patterns even when born full-term [214]. The combination of impaired or delayed secretory activation and an infant with early postnatal ineffective and/or inefficient sucking (see Text Box 2) is often associated with the failure to achieve a thriving breastfeeding dyad, resulting in partial or full reliance on formula.

Similar to pre-eclampsia, metabolomic differences including decreased concentration of proteins involved in glucose homeostasis also are observed in human milk from parents with gestational diabetes and/or insulin resistance compared to those with uncomplicated pregnancies [210]. How these differences may influence postnatal growth and gastrointestinal and metabolic development of the offspring is not known, but it raises concern for infants born to parents with these phenotypes.

#### Hunger-satiety signaling

Infants born with elevated fat mass, commonly with the phenotype of LGA at birth, especially when related to fetal exposure to diabetes from the birthing parent; insulin resistance; or obesity may demonstrate aberrations in appetite hormones and oral feeding ability at birth [214]. Potentially, these abnormalities are protective mechanisms to regulate the postnatal growth and body composition with the potential for what is described as catch-down growth [215, 216]. Identification of the effects specific to fetal exposures is complex since appetite hormones in the milk also are influenced by obesity in the birthing parent [208].

Differentiation of the impacts of phenotypic variation in the infant milk removal ability and metabolic signaling as influenced by fetal vs. postnatal milk composition exposures is clearly complex and represents an example of interacting systems.

The preceding section emphasizes the multiple considerations for optimal use of human milk for infants for whom gestation has been disrupted by shortened duration, by the robustness of the fetal environment, or by medical conditions of the lactating parent. In addition to infants born preterm, SGA, or LGA, infants with other conditions such as genetic and/or neurologic conditions may also exhibit impaired milk extraction and robust secretory activation. Current understanding of the parental factors and drivers of complex milk composition are discussed in other reports from WGs 1 and 2 [7, 155]. Selected research gaps related to the disruptions in the gestation and the ecology of infant inputs, milk composition, and lactation are summarized in Text Box 8.

Text Box 8 **Disruptions in gestation and the ecology of infant inputs, milk composition, and lactation: selected research gaps.**

• Are there effects on lactation regulation and milk composition specific to infants born at risk for growth and metabolic sequalae (e.g., preterm, SGA, and LGA)? If so, what is the mechanism?
• Do differences in gut maturity and function impact the response to exposures through human milk? Does the response to exposure differ according to the source of the milk (lactating parent or milk donor)?
• Are disruptions in gestation also linked to disruptions in secretory differentiation and postbirth lactation processes? Can effective and efficient milk removal postbirth mitigate these disruptions?
• Is development of the infant’s biological clock impacted by disruptions in the chronobiology of human milk?
• Does infant sex alter milk composition?
• What is the ecology of infant’s milk removal ability, metabolic programming induced by fetal exposures and/or by postnatal exposures, and milk composition?
• What novel biomarkers could detect the nutritional status and/or milk components specific to medically fragile infants (preterm, SGA, LGA, and other health conditions)?

Alt-text: Text Box 8

## Conclusions

The essential role of infants in the ecology of human lactation is indisputable, including, first and foremost, the establishment of effective, efficient, and comfortable milk removal, which is fundamental for the regulation of lactation processes. To this end, a comprehensive understanding of the biological and behavioral components of milk removal and accurate measurement of these components are essential. Optimal milk removal is fostered by the intimate bidirectional chemosensory communication between the birthing parent, starting in utero and extended to the young infant. Diet and xenobiotic exposures of the lactating parent, due to lifestyle choices or necessitated by medical treatments, affect not only milk production and milk composition but also the infant’s biological responses, either beneficially or adversely. Developing alongside the chemosensory signaling is the seeding and maturation of the infant microbiome, which transfers and exchanges with that of the parent and of the milk, forming additional bidirectional linkages.

As in any complex biological system, many points of potential disruption can and do occur, as illustrated by the effects of shortened or pathologically altered gestation on newborn and young infants. Even if anticipated, such potential disruptors can adversely impact the prospects for successful lactation and, simultaneously, the likelihood of meeting the nutritional and developmental needs of infants.

The use of human milk (parent’s own milk or donor human milk) is increasingly prioritized in hospital settings, including NICUs. These practices should be incorporated into clinical care guidelines with due considerations of the issues regarding translation of evidence to the policy covered by a separate report [102] and to realize their benefits while balancing the challenges presented by medical, nutritional, and developmental needs. The unique value of human milk and breastfeeding for immediate and long-term infant and parental health and wellbeing is increasingly recognized. To assure that the robust ecology of lactation is promoted and protected, appropriate consideration must be directed to the myriad of infant inputs that can either negate or profoundly strengthen that ecology.
